# Evaluation of a Targeted Next-Generation Sequencing Panel for the Non-Invasive Detection of Variants in Circulating DNA of Colorectal Cancer

**DOI:** 10.3390/jcm10194487

**Published:** 2021-09-29

**Authors:** Aitor Rodríguez-Casanova, Aida Bao-Caamano, Ramón M. Lago-Lestón, Elena Brozos-Vázquez, Nicolás Costa-Fraga, Isabel Ferreirós-Vidal, Ihab Abdulkader, Yolanda Vidal-Insua, Francisca Vázquez Rivera, Sonia Candamio Folgar, Rafael López-López, Laura Muinelo-Romay, Angel Diaz-Lagares

**Affiliations:** 1 Cancer Epigenomics, Translational Medical Oncology Group (Oncomet), Health Research Institute of Santiago (IDIS), University Clinical Hospital of Santiago (CHUS/SERGAS), 15706 Santiago de Compostela, Spain; aitorrodriguez@me.com (A.R.-C.); aida.bc11@gmail.com (A.B.-C.); nicocostafraga@gmail.com (N.C.-F.); 2 Translational Medical Oncology Group (Oncomet), Roche-Chus Joint Unit, Health Research Institute of Santiago (IDIS), 15706 Santiago de Compostela, Spain; rafael.lopez.lopez@sergas.es; 3 Universidade de Santiago de Compostela (USC), 15782 Santiago de Compostela, Spain; 4 Translational Medical Oncology Group (Oncomet), Liquid Biopsy Analysis Unit, Health Research Institute of Santiago (IDIS), 15706 Santiago de Compostela, Spain; Ramon.Manuel.Lago.Leston@sergas.es (R.M.L.-L.); isafv@hotmail.com (I.F.-V.); lmuirom@gmail.com (L.M.-R.); 5 Translational Medical Oncology Group (Oncomet), Health Research Institute of Santiago (IDIS), University Clinical Hospital of Santiago (CHUS/SERGAS), 15706 Santiago de Compostela, Spain; Elena.Maria.Brozos.Vazquez@sergas.es (E.B.-V.); Yolanda.Vidal.Insua@sergas.es (Y.V.-I.); Francisca.Vazquez.Rivera@sergas.es (F.V.R.); sonia.candamio.folgar@sergas.es (S.C.F.); 6 Department of Pathology, University Clinical Hospital of Santiago (CHUS/SERGAS), 15706 Santiago de Compostela, Spain; Ihab.Abdulkader.Nallib@sergas.es; 7 Centro de Investigación Biomédica en Red Cáncer (CIBERONC), 28029 Madrid, Spain

**Keywords:** colorectal cancer, TruSight Tumor 170, NGS, BEAMing, liquid biopsy, tumor biomarkers

## Abstract

Molecular profiling of circulating cell-free DNA (cfDNA) has shown utility for the management of colorectal cancer (CRC). TruSight Tumor 170 (TST170) is a next-generation sequencing (NGS) panel that covers 170 cancer-related genes, including *KRAS*, which is a key driver gene in CRC. We evaluated the capacity of TST170 to detect gene variants in cfDNA from a retrospective cohort of 20 metastatic CRC patients with known *KRAS* variants in tumor tissue and in cfDNA previously analyzed by pyrosequencing and BEAMing, respectively. The cfDNA of most of the patients (95%) was successfully sequenced. We frequently detected variants with clinical significance in *KRAS* (79%, 15/19) and *PIK3CA* (26%, 5/19) genes. Variants with potential clinical significance were also identified in another 27 cancer genes, such as *APC*. The type of *KRAS* variant detected in cfDNA by TST170 showed high concordance with those detected in tumor tissue (77%), and very high concordance with cfDNA analyzed by BEAMing (94%). The variant allele fractions for *KRAS* obtained in cfDNA by TST170 and BEAMing correlated strongly. This proof-of-principle study indicates that targeted NGS analysis of cfDNA with TST170 could be useful for non-invasive detection of gene variants in metastatic CRC patients, providing an assay that could be easily implemented for detecting somatic alterations in the clinic.

## 1. Introduction

Colorectal cancer (CRC) is the third most frequent cancer worldwide and the second leading cause of cancer mortality [1]. To date, certain gene alterations have been identified in metastatic colorectal cancer (mCRC) as clinically useful biomarkers. Among these, variants in specific codons of the Kirsten RAS (*KRAS*) oncogene are of particular interest due to their ability to predict tumor response to anti-epidermal growth factor receptor (*EGFR*)-targeted therapies [2]. In addition, other genetic alterations in relevant genes, such as *NRAS*, *BRAF*, and *PIK3CA,* are associated with mCRC [3].

Liquid biopsy has emerged in recent years as a non-invasive method for analysis of the molecular landscape of solid tumors using different types of biological fluids, including blood [4,5,6,7]. One of the most common strategies is analysis of circulating cell-free DNA (cfDNA) present in blood to detect tumor-specific alterations in the fraction originating from tumor cells (called circulating tumor DNA (ctDNA)) [8]. Molecular profiling of cfDNA in liquid biopsies can be performed by several strategies, including digital PCR (dPCR) and next-generation sequencing (NGS). In this sense, BEAMing (Beads, Emulsions, Amplification, and Magnetics) represents a highly sensitive dPCR method for identifying and quantifying hotspot variants in cancer-related genes of cfDNA, such as *KRAS* and *NRAS* [9,10]. This technology was used in the first assay clinically validated for determining the mutation status of *KRAS* in CRC (the OncoBEAM RAS CRC test, Sysmex Inostics) [11]. However, a limitation of dPCR is that it is unable to analyze a great number of genes in the same assay, which can be solved by NGS approaches [12,13].

Targeted NGS represents a reliable technology for characterizing tumors with the potential to screen for large cancer gene panels in both tissue and cfDNA samples. In this sense, some recent works have demonstrated the utility of using this type of approach to detect gene variants in CRC patients [14,15,16]. The use of NGS for cfDNA analyses may facilitate the detection of driver genes and provide valuable information on tumor heterogeneity and clonal evolution. In addition, this approach may reveal novel therapeutic targets for the application of personalized therapy and represents a promising tool for the management of CRC patients [15].

The TruSight Tumor 170 (TST170, Illumina) is an enrichment-based targeted next-generation sequencing (NGS) panel that covers the coding regions of 170 cancer-related genes. DNA analysis with TST170 allows for the detection of somatic variants (single nucleotide variants (SNVs), insertions/deletions (indels), and copy number variations (CNVs)). TST170 has been shown to be useful for molecular profiling of tumor tissues [17,18] and it covers a different set of cancer genes with respect to the other available targeted NGS assays previously used in CRC [15]. To our knowledge, TST170 has not been used before to address cfDNA characterization in cancer patients. The use of this assay in cfDNA would open the possibility of having a new non-invasive tool for the study of gene variants in cancer research or in a clinical setting. Therefore, the aim of this study was to evaluate the capacity of the TST170 panel to detect gene variants in cfDNA of mCRC patients. To achieve this aim, cfDNA of a retrospective cohort of mCRC patients with known *KRAS* variants in tumor tissue and cfDNA was analyzed by NGS with the TST170 panel. Collectively, the results obtained in this study indicate that cfDNA can be assayed by TST170 to identify cancer-associated gene variants in mCRC patients. This non-invasive approach could contribute to improving cancer research and precision oncology.

## 2. Materials and Methods

### 2.1. Study Participants

Patients were recruited for this retrospective study between September 2016 and January 2019 in the Medical Oncology Department at the University Clinical Hospital of Santiago de Compostela (CHUS), Spain, after signing the pertinent informed consent form approved by the Galician Ethical Committee (Ref. 2015/746). The cohort was composed of 20 patients diagnosed with mCRC and with known *KRAS* variants present in plasma cfDNA analyzed by BEAMing.

### 2.2. Blood and Tissue Samples

Blood samples were collected before therapeutic intervention in 10 mL Cell-Free DNA BCT tubes (Streck, La Vista, NE, USA), and processed within the next 3 days. Plasma was obtained after centrifugation of blood samples at 1600× *g* for 10 min followed by centrifugation at 6000× *g* for 10 min. Plasma was stored at −80 °C until use. All tumor tissues used for diagnoses were obtained according to standard-of-care (SOC) procedures.

### 2.3. Isolation of cfDNA from Plasma

DNA extraction from 1–3 mL of plasma was performed using a QIAamp Circulating Nucleic Acid Kit (Qiagen, Hilden, Germany) following the manufacturer’s protocol. cfDNA was eluted in 75 μL of kit-supplied elution buffer. Concentration was assessed using a Qubit 4.0 Fluorometer (Thermo Fisher Scientific, Waltham, MA, USA) with a Qubit™ dsDNA HS Assay Kit (Thermo Fisher Scientific) before sample storage at −20 °C.

### 2.4. Analysis of KRAS Variants in Tumor Tissues

The profile of *KRAS* variants in tumor tissues was analyzed in FFPE samples. Quantitative detection of *KRAS* variants in codons 12, 13, and 61 in genomic DNA was analyzed with a Therascreen^®^ *KRAS* Pyro^®^ Kit by pyrosequencing in a PyroMark^®^ Q24 system (Qiagen) according to the manufacturer’s recommendations.

### 2.5. Detection of Gene Variants in cfDNA by Digital PCR

*KRAS/NRAS* hotspot variants were analyzed in cfDNA by BEAMing using the OncoBEAM™ RAS CRC IVD assay (Sysmex Inostics, Hamburg, Germany) as previously described [19]. *KRAS* variants (p.G12D, p.G12S, p.G13D) were also analyzed by droplet digital PCR (ddPCR) following the manufacturer’s recommendations in a QX200 System (Bio-Rad, Hercules, CA, USA) (Table S1). Specific gBlocks Gene Fragments (Integrated DNA Technologies, Coralville, IA, USA) were used as positive controls. The percentage of variant allele fractions (VAFs) was calculated as the fractional abundance of variant alleles with respect to wild-type (WT) alleles.

### 2.6. Analysis of cfDNA by Targeted NGS with TST170

The list of genes detected by TST170 is shown in Table S2. cfDNA libraries for sequencing were prepared using a TST170 kit (Illumina, San Diego, CA, USA) following the manufacturer’s reference guides. For library preparation, 35–100 ng of cfDNA was used (Table S3). As a quality control, libraries were made using 40 and 100 ng of Structural Multiplex cfDNA Reference Standard (Horizon Discovery, Waterbeach, UK). The workflow recommended for TST170 was followed, omitting the shearing step. Libraries were sequenced in a NextSeq 500 (Illumina) and data analyzed on BaseSpace Sequence Hub (Illumina). Variant calling was performed using the TST170 v2.0 app, which is a Docker-based software package that analyzes sequencing reads from libraries prepared with the TST170 sequencing panel. For variant interpretation, the cloud-based interpretation and reporting platform Variant Interpreter v2.9 (Illumina) was used. FastQ files were aligned to the human reference genome (Human, UCSC hg19) with the TST170 app using the Burrows–Wheeler aligner (BWA). Variants were processed by BaseSpace Variant Interpreter; variant calling was performed using the variant caller Pisces v5.2, and variants were annotated using the BaseSpace Annotation Engine v3.1. The default software quality filter was used to exclude low-confidence variants. The clinical significance of variants according to the American College of Medical genetics and genomics (ACMG) and the Association for Molecular Pathology (AMP) was evaluated using the somatic option of VarSome Clinical 9.4 (Saphetor).

### 2.7. Statistical Analysis

Quantitative variables are shown as means ± standard deviation (SD), and frequencies as percentages (%). Linear regression and Bland–Altman analysis were performed for comparison between methods. Differences between groups were assessed with Student’s t-test according to the normality of the distribution. A *p*-value < 0.05 was considered statistically significant. All statistical analyses were performed using GraphPad Prism 8.0 (GraphPad Software, La Jolla, CA, USA). The sensitivity (S) and specificity (Sp) of TST170 to detect gene variants in cfDNA were determined according to the following calculations: S (%) = (TP/TP + FN) × 100 and Sp (%) = (TN/TN + FP) × 100, where TP, FN, TN, and FP represent true positives, false negatives, true negatives, and false positives, respectively.

## 3. Results

### 3.1. Patient Characteristics

To evaluate the capacity of TST170 to detect gene variants in cfDNA, we selected a cohort of 20 mCRC patients with known *KRAS* variants, which is one of the genes included in the TST170 panel and previously analyzed in cfDNA by BEAMing. In particular, all the selected patients of this cohort had *KRAS* variants in cfDNA, and most of them also had *KRAS* variants in the corresponding paired tumor tissues. The clinical characteristics of the patients are displayed in Table 1 and Table S4. Six patients were female and 14 males. The average age was 65 ± 15 years. Most of the patients had tumors with adenocarcinoma histology and left colon or rectum localization and had not received prior systemic therapy before plasma collection.

### 3.2. Analysis of Variants in cfDNA of mCRC Patients Using the TST170 Targeted Panel

To evaluate the feasibility of using TST170 to detect variants in cfDNA, a total of 20 mCRC patients were analyzed. As a control, we used a cfDNA reference standard, which contains validated variants, including SNVs, indels, and CNVs. Importantly, the TST170 panel was able to detect all variants expected in the control (Table S5). Among the whole mCRC cohort, only one patient’s sample yielded a mean coverage <500× with the TST170 assay. This patient’s sample was not considered for further analysis. The cfDNA analysis of the other 19 patients by TST170 provided high-depth sequencing with a mean coverage of ~2500× (range: 620×–4595×).

**Figure 1:**
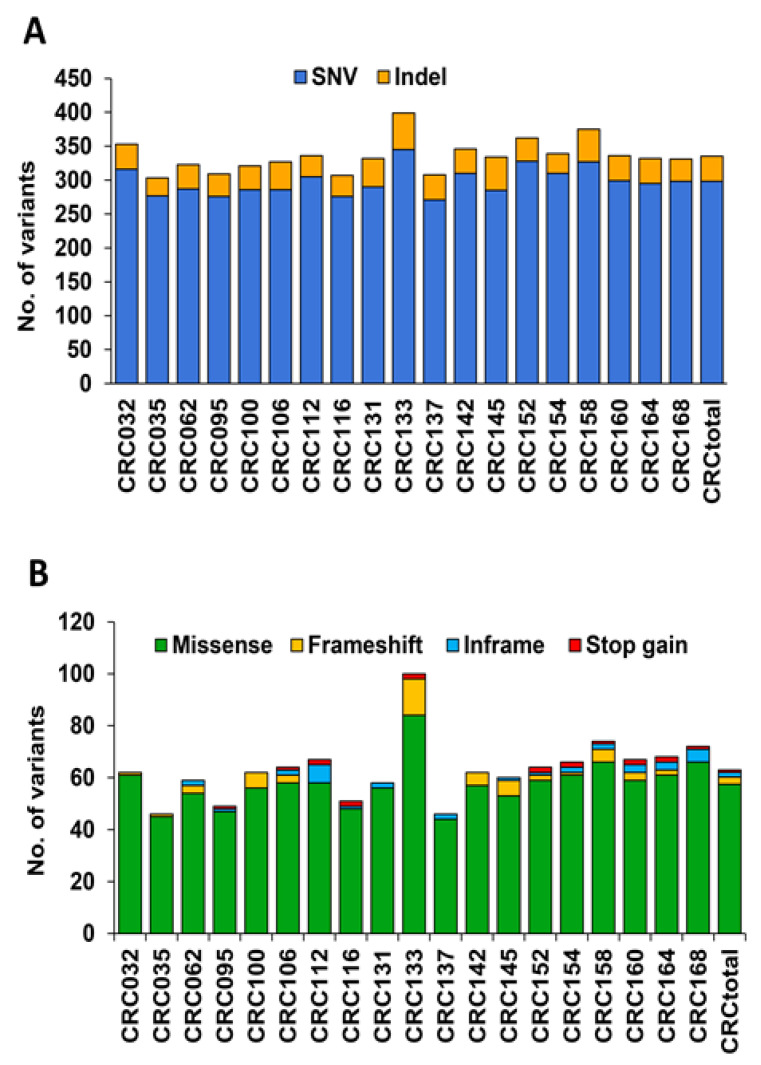
Overview of the variant distribution detected in cfDNA of CRC patients by TST170. (**A**) Global distribution of SNVs and indels in mCRC patients. (**B**) Distribution of SNVs and indels in mCRC patients according to variant type. The term “CRCtotal” represents the mean for all CRC patients analyzed of total variants detected per patient.

TST170 detected SNVs and indels in the cfDNA of all patients analyzed (Figure 1A). Among the observed variants, we detected frameshift, inframe, missense, and stop gain variants (Figure 1B, Tables S6 and S7), but these were not evenly distributed among patients. Although all patients showed more than one of these types of variants, some patients (37%, 7/19) displayed two types of variants, some patients (26%, 5/19) displayed three types, and others (37%, 7/19) displayed four types. Missense variants were the most frequent, and the only type present in all patients. On the other hand, the analysis of cfDNA by TST170 was also able to identify CNVs in 1 out of the 19 patients analyzed (5%, 1/19) (Table S8).

### 3.3. Identification of Cancer-Associated Variants with Clinical Significance in cfDNA of mCRC Patients

**Figure 2:**
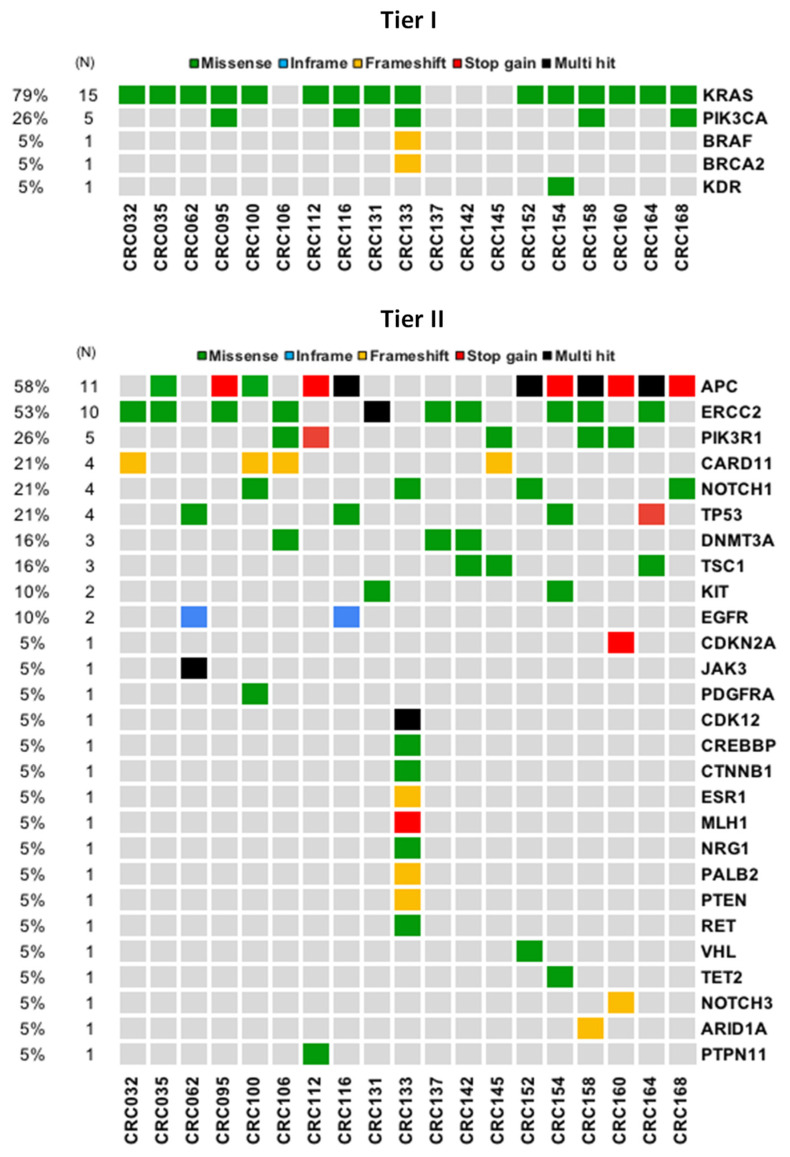
Spectrum of variants in cfDNA of CRC patients according to the cancer-associated clinical significance. The oncoplots show the frequency and distribution of the tier I and II variants detected in cfDNA of CRC patients. “Multi hit” indicates that more than one variant in a gene was found in the same patient. The number (*N*) and percentage of patients is shown on the left.

One of the key challenges in precision oncology is the identification and pathological interpretation of cancer-associated variants detected by sequencing [20]. Thus, to evaluate the pathogenicity of the variants detected in cfDNA by TST170, we used the somatic option of the in silico pipeline from VarSome Clinical, which follows the four-tier system recommended by the American College of Medical Genetics and Genomics (ACMG) and the Association for Molecular Pathology (AMP). This system classifies somatic variants into four categories based on their clinical impact: tier I, variants with strong clinical significance; tier II, variants with potential clinical significance; tier III, variants with unknown clinical significance; and tier IV, benign or likely benign variants [20]. Using this approach, we observed that 0.4% of all the variants detected in cfDNA of our cohort by TST170 were classified as tier I and 1.5% as tier II, while 3.8% were considered tier III and 94.3% were tier IV (Figure S1). Importantly, we frequently detected tier I variants in *KRAS* (79%, 15/19) and *PIK3CA* (26%, 5/19) genes of our CRC cohort. In addition, tier II variants were detected in another 27 cancer-relevant genes, including *APC*, which was the gene with the most tier II variants identified (Figure 2).

### 3.4. Analysis of KRAS Variants in cfDNA of mCRC Patients and Concordance with Tissue Analysis

**Figure 3:**
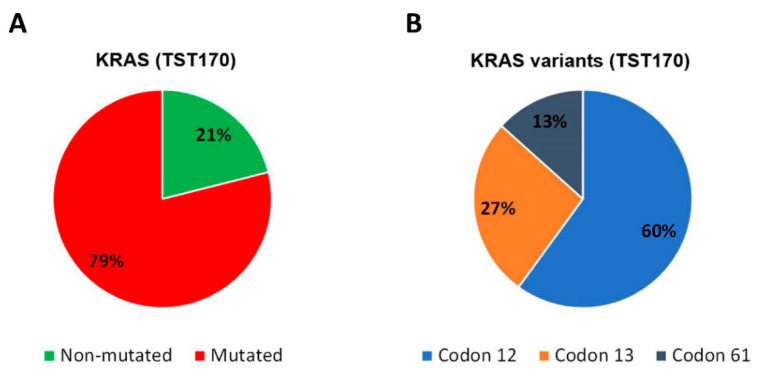
Distribution of *KRAS* variants detected in cfDNA of mCRC patients (*N* = 19) by TST170. (**A**) Frequency of CRC patients with *KRAS* variants detected in cfDNA by TST170. (**B**) Frequency of *KRAS* variants detected in cfDNA by TST170 according to codon location.

The genetic status of *KRAS* is clinically relevant in CRC patients [2]; therefore, to verify the ability of TST170 to detect gene variants in cfDNA, we focused this study on this gene. Among the *KRAS* variants identified in cfDNA of our cohort by TST1170, nine (60%) were located in codon 12, four (27%) in codon 13, and two (13%) in codon 61 (Figure 3).

A high correlation between *KRAS* variants detected in tumor tissue and cfDNA of CRC patients has previously been reported [21,22]. Table 2 summarizes, for each mCRC patient, the *KRAS* variant detected in tumor tissues by pyrosequencing and in cfDNA by BEAMing or TST170 assays. TST170 analysis of cfDNA was able to detect *KRAS* variants in 81% (13/16) of the 16 patients with available information on the genetic status of *KRAS* in their tumor tissue (Table 2). Importantly, 77% (10/13) of the *KRAS* variants detected in cfDNA by TST170 were the same as those found in tumor tissue, showing high concordance between both analyses. In addition, considering the *KRAS* codon number altered, the concordance between cfDNA analysis by TST170 and the genetic status of tumor tissue increased to 92% (12/13) (Table 2). As expected, we obtained a similarly high correlation (15/16, 94%) when we compared the available *KRAS* variants in tumor tissue with the results of cfDNA analysis by BEAMing (Table 2), which is an extensively validated method for assessing *KRAS* variants in the cfDNA of mCRC patients [19,23].

### 3.5. Concordance of KRAS Variants in cfDNA of mCRC Patients by BEAMing and TST170

Since BEAMing is a reference assay for analysis of the status of *KRAS* in the cfDNA of CRC patients [19,23], we compared the *KRAS* variants obtained in cfDNA by this method with those obtained by TST170 (Table 2).

**Figure 4:**
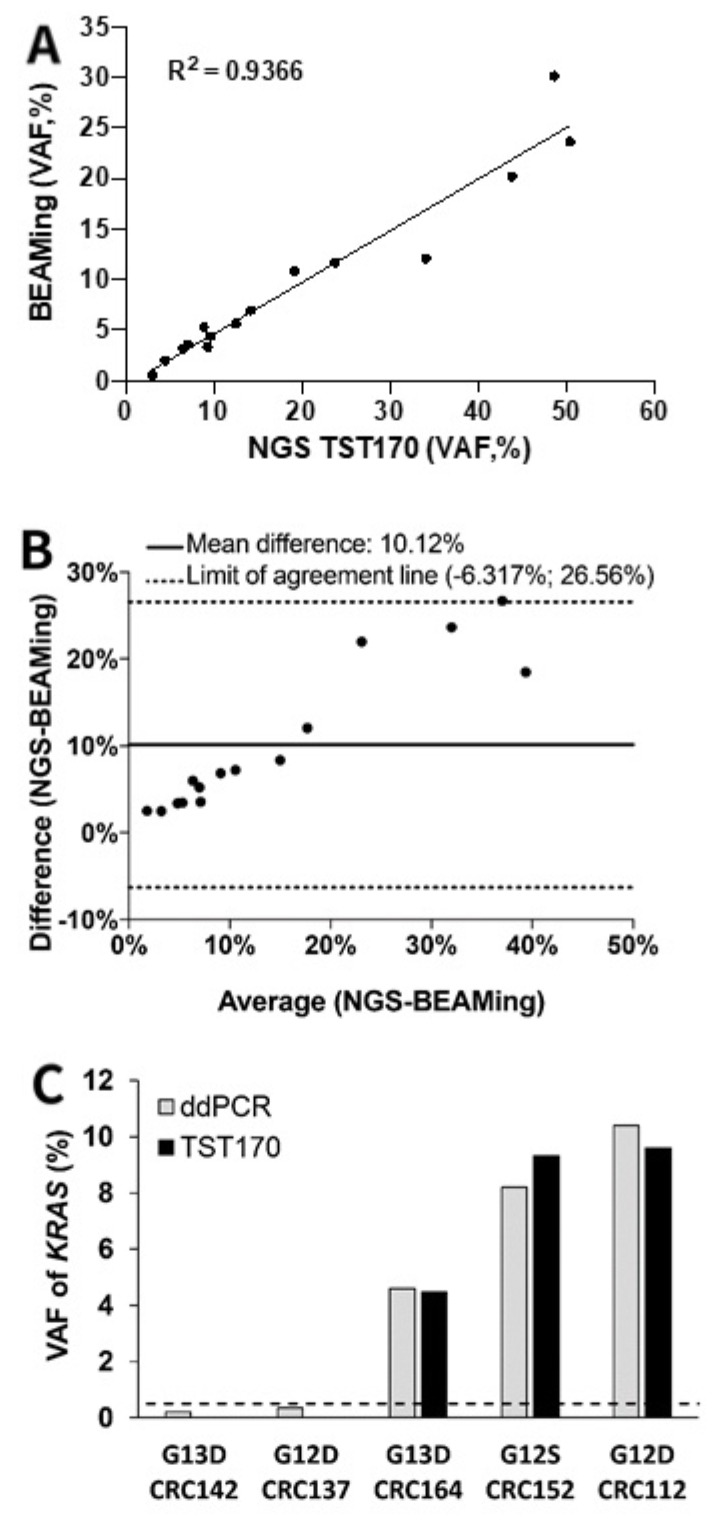
TST170 and digital PCR analysis for *KRAS* variants in cfDNA. (**A**) Linear regression analysis of *KRAS* VAFs in cfDNA analyzed by TST170 and BEAMing. (**B**) Bland–Altman plot of the *KRAS* VAFs between TST170 and BEAMing. (**C**) Validation of the *KRAS* status by ddPCR in cfDNA samples with discordant results between TS170 analysis and BEAMing or tumor tissue status. The dotted line represents a VAF ≥ 0.5%.

TST170 was able to detect *KRAS* variants in patients in whom the variant allele fractions (VAFs) obtained with BEAMing were ≥0.5%. Therefore, we used this value of VAF as the limit of detection (LOD) to perform comparative analysis between TST170 and BEAMing. Importantly, TST170 detected the *KRAS* variants in the same codon as BEAMing in 15 of the 16 patients analyzed, showing a high sensitivity (94%) to detect *KRAS* variants. In addition, linear regression analysis showed a high correlation (R^2^ = 0.9366, y = −0.49 + 0.51x) between *KRAS* VAFs obtained by BEAMing and TST170 (Figure 4A). To quantitate the agreement between both methods, we performed a Bland–Altman analysis, which showed a mean difference of 10.12% between BEAMing and TST170 (Figure 4B).

In addition to evaluating the sensitivity of TST170 and its concordance with the BEAMing assay, we also decided to evaluate the specificity of TST170 for cfDNA analysis. Since all patients of our cohort were *KRAS* mutated in cfDNA by BEAMing, we evaluated the specificity of TST170 based on the status of *NRAS*, another relevant gene for CRC that was WT in cfDNA by BEAMing analysis for most of the patients of our cohort (Table 1). Thus, considering the LOD of TST170 previously observed in our work for cfDNA analysis (VAF ≥ 0.5%), TST170 detected the WT status of *NRAS* in the cfDNA of all the patients (14/14) of our cohort, showing a specificity for cfDNA analysis of 100% (Table S9).

### 3.6. Description of KRAS Variants in Discordant Samples Detected in cfDNA by TST170

In some patients, the analysis of *KRAS* variants in cfDNA with TST170 showed some discordance with respect to the cfDNA analyzed by BEAMing or the status of tumor tissues. To verify whether these discordances were due to limitations of TST170, we performed a reanalysis of the available cfDNA samples using a *KRAS*-specific droplet digital PCR (ddPCR) assay. TST170 was unable to detect *KRAS* variants in three patients (CRC106, CRC137, CRC142) in whom these alterations were detected both by BEAMing and tissue analysis (Table 2). In these samples, *KRAS* showed a VAF < 0.5% by BEAMing, which is the LOD obtained in our cohort to detect *KRAS* variants by TST170. *KRAS* reanalysis of two of the cfDNA samples (CRC137 and CRC142) by ddPCR detected the expected *KRAS* variants with a VAF < 0.5% (Figure 4C), confirming the low allele fraction as the cause for the discordancy. In three patients (CRC152, CRC160, CRC164), tumor tissue showed variants in the same *KRAS* codon number as was seen in cfDNA analyzed by BEAMing and TST170; however, the specific variant identified was different between TST170 and tumor tissue (Table 2). Importantly, we reanalyzed the cfDNA of two of these three patients (CRC152, CRC164) by ddPCR and identified the same *KRAS* variant as TST170 (Figure 4C). Furthermore, two patients (CRC112, CRC133) without *KRAS* genetic status available in tumor tissue showed the same codon of *KRAS* altered after the analysis with both BEAMing and TST170 (Table 2). Importantly, reanalysis of one patient by ddPCR (CRC112) confirmed the type of variant identified by TST170 (Figure 4C).

### 3.7. KRAS Analysis in cfDNA According to Clinical–Pathological Characteristics of Patients

Despite the limited size of the patient cohort for finding clear statistically significant associations, we explored the potential relationship of *KRAS* variants identified by TST170 and BEAMing with clinical–pathological characteristics of patients. Both TST170 and BEAMing showed lower *KRAS* VAFs in patients without liver metastasis than in those with this type of metastasis (Figure S2). According to these results, none of the four patients with *KRAS* variants detected by BEAMing but undetected with TST170 showed liver metastasis. In particular, of these four cases, two of them had lung metastasis, and the other two cases showed only peritoneal affectation. In addition, both TST170 and BEAMing showed lower *KRAS* VAFs in patients with previous primary tumor resection than those without resection (Figure S2). In fact, all BEAMing/TST170 discordant cases had previously undergone primary tumor resection.

## 4. Discussion

Approaches based on targeted NGS have been demonstrated to be useful for the detection of gene variants in tumor tissues and cfDNA of several tumor types, including CRC [14,16]. The use of new NGS assays in liquid biopsy is a very relevant approach for the non-invasive management of CRC patients, which can facilitate precision medicine strategies with clinical benefits for oncological practice [15]. In this study, we analyzed a cohort of mCRC patients with known *KRAS* variants in both tissue and plasma to investigate the performance of the targeted NGS panel TST170 in detecting gene variants in cfDNA. TST170 covers the coding regions of 170 cancer-related genes and has been successfully used in tumor tissues to characterize genetic alterations [17,18]. The use of this assay in cfDNA may provide a new non-invasive tool for the study of gene variants in cancer research or in a clinical setting without the need of a specific design for cfDNA. To our knowledge, this is the first study that evaluates the TST170 panel on cfDNA from cancer patients. The results obtained in this work demonstrate the feasibility of using TST170 to detect gene variants in cfDNA. Thus, using this NGS panel in cfDNA of mCRC patients and following ACMG and AMP guidelines, we were able to frequently identify cancer-associated variants with strong clinical significance in relevant genes, such as *KRAS* and *PIK3CA*. In addition, we also identified variants with potential clinical significance in another 27 cancer-related genes. Of note, the *KRAS* variants identified in cfDNA by TST170 showed high concordance with tumor tissue and cfDNA analyzed by BEAMing. This proof-of-principle study indicates that cfDNA can be assayed by TST170 to identify the presence of clinically relevant variants in mCRC patients, representing an alternative non-invasive approach that could be useful in cancer research and in the clinic, contributing to solving some of the limitations of tumor tissue biopsies [24,25].

In the present work, the TST170 assay was especially useful for identification of SNVs and indels in cfDNA, as these variants were detected in all CRC patients analyzed. Importantly, many of the variants detected were frameshift, inframe, missense, and stop gain variants. These types of variants are associated with the capability of producing clinically relevant effects in genes driving cancer progression [26]. Therefore, detection of these variants in our work supports the possibility of using the TST170 panel, not only in tissue samples, but also in cfDNA in a research or clinical setting. Of note, missense variants are among the most frequently observed alterations in CRC [27,28,29]. In accordance with this, missense variants were the most frequently detected type of alteration among all analyzed patients in our cohort. In contrast to the high frequency of SNVs and indels identified, analysis of cfDNA with TST170 only detected CNVs in one CRC patient. The low number of CNVs detected could be due to: (i) this panel’s capacity to detect CNVs in a small subset of genes with respect to SNV/indel genes; (ii) the small size of the cohort analyzed; or (iii) the lower overall frequency of CNVs in the human genome, making their detection more technically challenging than SNVs and indels [30,31].

Importantly, as expected, the analysis of cfDNA by TST170 in our cohort was able to detect frequent *KRAS* variants with strong clinical significance. In addition, this assay also detected variants in other relevant genes associated with cancer pathways and/or with clinical implications for CRC patients, such as *PIK3CA*, *BRAF, EGFR*, *APC,* and *TP53,* among others [28,32,33]. The high frequency of variants observed in some of these genes, such as for *PIK3CA*, could be influenced by the small size of our cohort and the high frequency of patients with *KRAS* variants [34]. In addition, we observed a low frequency of *BRAF* variants, which is in line with previous studies showing that concomitant variants in *BRAF* and *KRAS* rarely occur in CRC [35].

Detection of variants in specific codons of the oncogene *KRAS* is of particular interest in mCRC patients for its value to predict response to anti-*EGFR* targeted therapies [2]. Therefore, it is especially relevant to have non-invasive approaches to detect this type of variant in the clinic. BEAMing is a highly sensitive digital PCR assay considered the gold standard method for the genetic analysis of *KRAS* in cfDNA of CRC patients [19,23]. In this work, TST170 was able to detect *KRAS* variants in patients who had a value of VAF obtained by BEAMing of ≥0.5%, indicating that TST170 can successfully identify variants with VAFs ≥ 0.5%. This value of VAF represents the LOD for *KRAS* variants in our cohort and is in the range of analytical sensitivity reported in other studies for targeted NGS assays to evaluate cfDNA in cancer patients [11,36].

Analysis of cfDNA by TST170 was not able to detect any of the expected *KRAS* variants in 4 out of 19 patients evaluated in our study. Of note, these four patients presented lung or peritoneal metastasis without liver affectation. The lack of expected variant detection in these patients could be explained by the location of metastasis, since it has been recently reported that in mCRC both lung and peritoneal lesions, compared with other metastatic sites such as the liver, have significantly lower maximum allele frequencies and a lower number of detected variants, suggesting lower levels of ctDNA release as compared with other metastatic sites like the liver [10,37]. In addition, the lack of expected variant detection in these four patients could be influenced by the amount of cfDNA used for the TST170 assay, which was in these four cases at the limit of the manufacturer´s recommendations.

Importantly, TST170 was able to detect *KRAS* variants in the cfDNA of most of the patients (77%) who also had *KRAS* alterations in their tumor tissue. This result is in agreement with previous studies in CRC patients that showed a high correlation between the *KRAS* variants detected in their tumor tissues and cfDNA [21,22]. Relevantly, we obtained high concordance (94%) for detection of *KRAS* variants in cfDNA between TST170 and BEAMing. However, although there was a strong correlation between both methods, TST170 showed different values of *KRAS* VAFs than BEAMing. Similarly to this work, other authors found variations in *KRAS* VAF values by NGS and digital PCR approaches, but with a good correlation between both technologies [38]. Importantly, the few discordant cases observed between TST170 and BEAMing were patients without liver metastasis, and with previous primary tumor resection, for whom ctDNA shedding is well accepted to be low [10]. In these cases, TST170 could be more limited than BEAMing in detecting *KRAS* variants in cfDNA. However, TST170 was able to detect other gene variants in the discordant cases, reinforcing the interest of applying a more comprehensive assay to have a more global view of the disease. Besides, in all samples in which *KRAS* sequences showed discordance between tissue and TST170, reanalysis of cfDNA by ddPCR confirmed the results obtained by TST170, supporting the reliability of the data obtained with this NGS assay. The observed discordances in *KRAS* sequencing from tissue and TST170 could be explained by the heterogeneity and/or clonal evolution of tumors, which yield variable representation within the cfDNA of subclonal tumor cell populations [39]. The high correlation in the genetic status of *KRAS* obtained between cfDNA and matched tumor tissue suggests that the *KRAS* variants identified in the cfDNA of our patient cohort accurately represent the tumor tissue. However, the tumoral origin of the genetic variants in other genes found by our analyses of cfDNA should be interpreted with caution due to lack of data from matched tumor tissue or white blood cells (WBCs) [40].

This proof-of-principle study indicates that targeted NGS analysis of cfDNA with the TST170 panel could be useful for non-invasive detection of clinically relevant variants in liquid biopsy of mCRC patients. In future studies, analysis of cfDNA samples with unknown genetic variant status (blind samples) would be useful to obtain additional information on the capabilities of TST170.

## 5. Conclusions

Taken together, our data indicate that targeted NGS analysis of cfDNA with the TST170 panel could be useful for non-invasive detection of gene variants in metastatic CRC patients, providing an assay that could be easily implemented for detecting somatic alterations in the clinic. These data support further investigation into applications of this NGS approach to non-invasively characterizing the genetic landscape of tumors.

## Figures and Tables

**Table 1:** Clinical characteristics of mCRC patients included in the study.

Characteristics	Patients (*N* = 19)	
	No.	%
**Age (years)**		
<60	5	26
60–69	5	26
70–79	7	37
>80	2	11
**Gender**		
Female	6	32
Male	13	68
**Histology**		
Adenocarcinoma	18	95
Mucinous adenocarcinoma	1	5
**Primary tumor location**		
Right colon	8	42
Left colon/rectum	11	58
**Number of metastatic locations**		
1	9	47
≥2	10	53
**Metastatic location**		
Liver	10	53
Lung	10	53
Peritoneum	5	26
**Previously resected primary tumor**		
Yes	10	53
No	9	47
**Previous systemic treatment ^1^**		
Yes	6	32
No	13	68
**MSI status ^2^**		
Negative	16	84
Unknown	3	16
***KRAS* status in tissue**		
Wild type	1	5
Mutated	16	84
Unknown	2	11
***KRAS* status in cfDNA**		
Wild type	0	0
Mutated	19	100
***NRAS* status in cfDNA**		
Wild type	15	79
Mutated	1	5
Unknown	3	16
**Tissue biopsy location**		
Primary Tumor	14	74
Metastasis	2	10
Unknown	3	16

^1^ Surgery/systemic treatment before plasma collection. ^2^ MSI, microsatellite instability.

**Table 2:** *KRAS* variants detected in tumor tissues and cfDNA in mCRC patients (*N* = 19).

Sample ID	*KRAS* Variants		
	Tumor Tissue	cfDNA	
		BEAMing (VAF, %)	TST170 (VAF, %)
CRC032	p.Q61L	KR3Cdn61 (0.55)	p.Q61L (3.08)
CRC035	p.G12S	KR2Cdn12 (11.68)	p.G12S (23.72)
CRC062	p.G12V	KR2Cdn12 (23.68)	p.G12V (50.37)
CRC095	p.G12V	KR2Cdn12 (5.33)	p.G12V (8.87)
CRC100	p.G13D	KR2Cdn13 (6.96)	p.G13D (14.18)
CRC106	p.G12D	KR2Cdn12 (0.32)	ND
CRC112	NA	KR2Cdn12 (4.38)	p.G12D (9.6)
CRC116	p.G13D	KR2Cdn13 (30.12)	p.G13D (48.61)
CRC131	p.G12V	KR2Cdn12 (12.10)	p.G12V (34.09)
CRC133	NA	KR2Cdn13 (10.84)	p.G13D (19.19)
CRC137	p.G12D	KR2Cdn12 (0.21)	ND
CRC142	p.G13D	KR2Cdn13 (0.11)	ND
CRC145	NA	KR2Cdn12 (0.70)	ND
CRC152	p.G12D	KR2Cdn12 (3.35)	p.G12S (9.32) *
CRC154	p.Q61L	KR3Cdn61 (20.17)	p.Q61L (43.83)
CRC158	p.G12V	KR2Cdn12 (5.69)	p.G12V (12.53)
CRC160	p.G12D	KR2Cdn12 (3.59)	p.G12A (7.04) *
CRC164	WT	KR2Cdn13 (1.98)	p.G13D (4.48) *
CRC168	p.G12A	KR2Cdn12 (3.16)	p.G12A (6.53)

VAF, variant allele fraction; NA, not available; ND, not detected; KR2, *KRAS* exon 2; KR3, *KRAS* exon 3; Cdn12, codon 12; Cdn13, codon 13; Cdn61, codon 61. * *KRAS* variant detected in cfDNA with TST170 showing a discrepancy with tumor tissue.

## Data Availability

The data presented in this study are openly available in the Sequence Read Archive (SRA) at the National Center for Biotechnology Information (NCBI), reference number PRJNA761891.

## Supplementary Materials

The following are available online at https://www.mdpi.com/article/10.3390/jcm10194487/s1, Figure S1: Distribution of variants detected by TST170 in cfDNA of mCRC patients according to their clinical impact, Figure S2: Impact of *KRAS* VAFs detected by TST170 and BEAMing on patient clinical–pathological characteristics, Table S1: Characteristics of the *KRAS* assays used by ddPCR, Table S2: List of genes analyzed in DNA by the TST170 panel, Table S3: Quantity of cfDNA used for TST170 assay, Table S4: Tumor location of mCRC patients included in the study, Table S5: Analysis of variants in the reference standard cfDNA by TST170, Tables S6 and S7: List of genes with variants (frameshift, inframe, missense, stop gain) detected in cfDNA of mCRC patients by TST170, Table S8: CNVs detected in cfDNA of patient CRC100 by TST170, Table S9: *NRAS* status in cfDNA of mCRC patients analyzed by BEAMing and TST170.
