# Supplementary material for: Evaluation of a Targeted Next-Generation Sequencing Panel for the Non-Invasive Detection of Variants in Circulating DNA of Colorectal Cancer
Source: J Clin Med. 2021 Sep 29;10(19):4487. doi: 10.3390/jcm10194487 (PMC8509146; doi:10.3390/jcm10194487)
Supplement: Supplementary file 1 [file jcm-10-04487-s001.zip › jcm-1330826-supplementary.pdf]

Table S1. Characteristics of the *KRAS* assays used by ddPCR.

Gene	Assay ID	COSMIC ID	Expected variant	
			AA mutation	CDS mutation
KRAS	dHsaMDV2010001	COSM521	p.G12D	c.35G>A
	dHsaMDV2510588	COSM517	p.G12S	c.34G>A
	dHsaMDV2510598	COSM532	p.G13D	c.38G>A

AA, aminoacid; CDS, coding sequence.

Table S2. List of genes analyzed in DNA by the TST170 panel.

**SNVs and Indels**									
AKT1	*BRIP1*	*CREBBP*	*FANCI*	*FGFR2*	*JAK3*	*MSH3*	*PALB2*	*RAD51D*	*TSC1*
AKT2	*BTK*	*CSF1R*	*FANCL*	*FGFR3*	*KDR*	*MSH6*	*PDGFRA*	*RAD54L*	*TSC2*
AKT3	*CARD11*	*CTNNB1*	*FBXW7*	*FGFR4*	*KIT*	*MTOR*	*PDGFRB*	*RB1*	*VHL*
ALK	*CCND1*	*DDR2*	*FGF1*	*FLT1*	*KMT2A*	*MUTYH*	*PIK3CA*	*RET*	*XRCC2*
APC	*CCND2*	*DNMT3A*	*FGF10*	*FLT3*	*KRAS*	*MYC*	*PIK3CB*	*RICTOR*	*TSC1*
AR	*CCNE1*	*EGFR*	*FGF14*	*FOXL2*	*MAP2K1*	*MYCL1*	*PIK3CD*	*ROS1*	*TSC2*
ARID1A	*CD79A*	*EP300*	*FGF2*	*GEN1*	*MAP2K2*	*MYCN*	*PIK3CG*	*RPS6KB1*	
ATM	*CD79B*	*ERBB2*	*FGF23*	*GNA11*	*MCL1*	*MYD88*	*PIK3R1*	*SLX4*	
ATR	*CDH1*	*ERBB3*	*FGF3*	*GNAQ*	*MDM2*	*NBN*	*PMS2*	*SMAD4*	
BAP1	*CDK12*	*ERBB4*	*FGF4*	*GNAS*	*MDM4*	*NF1*	*PPP2R2A*	*SMARCB1*	
BARD1	*CDK4*	*ERCC1*	*FGF5*	*HNF1A*	*MET*	*NOTCH1*	*PTCH1*	*SMO*	
BCL2	*CDK6*	*ERCC2*	*FGF6*	*HRAS*	*MLH1*	*NOTCH2*	*PTEN*	*SRC*	
BCL6	*CDKN2A*	*ERG*	*FGF7*	*IDH1*	*MLLT3*	*NOTCH3*	*PTPN11*	*STK11*	
BRAF	*CEBPA*	*ESR1*	*FGF8*	*IDH2*	*MPL*	*NPM1*	*RAD51*	*TERT*	
BRCA1	*CHEK1*	*EZH2*	*FGF9*	*INPP4B*	*MRE11A*	*NRAS*	*RAD51B*	*TET2*	
BRCA2	*CHEK2*	*FAM175A*	*FGFR1*	*JAK2*	*MSH2*	*NRG1*	*RAD51C*	*TP53*	
**CNVs**									
AKT2	*BRCA2*	*CHEK1*	*ERCC2*	*FGF2*	*FGF7*	*FGFR4*	*MDM4*	*NRG1*	*RAF1*
ALK	*CCND1*	*CHEK2*	*ESR1*	*FGF23*	*FGF8*	*JAK2*	*MET*	*PDGFRA*	*RET*
AR	*CCND3*	*EGFR*	*FGF1*	*FGF3*	*FGF9*	*KIT*	*MYC*	*PDGFRB*	*RICTOR*
ATM	*CCNE1*	*ERBB2*	*FGF10*	*FGF4*	*FGFR1*	*KRAS*	*MYCL1*	*PIK3CA*	*RPS6KB1*
BRAF	*CDK4*	*ERBB3*	*FGF14*	*FGF5*	*FGFR2*	*LAMP1*	*MYCN*	*PIK3CB*	*TFRC*
BRCA1	*CDK6*	*ERCC1*	*FGF19*	*FGF6*	*FGFR3*	*MDM2*	*NRAS*	*PTEN*	

SNVs, single nucleotide variants; indels, insertion/deletion; CNVs, copy number variants.

Table S3. Quantity of cfDNA used for TST170 assay.

Sample ID	cfDNA (ng)
CRC032	40
CRC035	50
CRC062	100
CRC095	35
CRC100	40
CRC106	35
CRC112	100
CRC116	100
CRC131	100
CRC133	100
CRC137	40
CRC142	40
CRC145	35
CRC152	40
CRC154	40
CRC158	40
CRC160	40
CRC164	40
CRC168	40

Table S4. Tumor location of mCRC patients included in the study.

Sample ID	Primary tumor location	Metastasis location				Tissue biopsy location
		Liver	Lung	Peritoneal	Lymph Node	
**CRC032**	Right Colon	Yes	No	No	No	Primary
**CRC035**	Right Colon	No	No	Yes	No	Primary
**CRC062**	Left Colon/rectum	Yes	Yes	No	No	Primary
**CRC095**	Left Colon/rectum	Yes	Yes	No	No	Primary
**CRC100**	Right Colon	No	Yes	Yes	Yes	Metastasis (lymph node)
**CRC106**	Right Colon	No	Yes	No	Yes	Primary
**CRC112**	Right Colon	Yes	No	No	No	Unknown
**CRC116**	Left Colon/rectum	Yes	No	No	No	Primary
**CRC131**	Left Colon/rectum	Yes	Yes	No	No	Primary
**CRC133**	Right Colon	Yes	No	No	No	Unknown
**CRC137**	Left Colon/rectum	No	No	Yes	No	Primary
**CRC142**	Left Colon/rectum	No	Yes	No	Yes	Metastasis (lung)
**CRC145**	Left Colon/rectum	No	No	Yes	No	Unknown
**CRC152**	Left Colon/rectum	No	Yes	No	Yes	Primary
**CRC154**	Left Colon/rectum	Yes	Yes	No	No	Primary
**CRC158**	Left Colon/rectum	Yes	Yes	No	No	Primary
**CRC160**	Left Colon/rectum	No	No	No	Yes	Primary
**CRC164**	Right Colon	No	Yes	Yes	Yes	Primary
**CRC168**	Right Colon	Yes	No	No	Yes	Primary

Table S5. Analysis of variants in the reference standard cfDNA by TST170.

Gene	Variant type	Expected variant	Expected VAF (%)	Detected variant		Detected VAF (%)	
				40 ng DNA	100 ng DNA	40 ng DNA	100 ng DNA
GNA11	SNV	p.Q209L	5.6	p.Q209L		5.5	4.6
AKT1	SNV	p.E17K	5.0	p.E17K		4.2	3.6
PIK3CA	SNV	p.E545K	5.6	p.E545K		5.0	4.3
EGFR	Indel	p.V769_D770insASV	5.6	p.V769_D770insASV		2.9	2.8
EGFR	Indel	p.E746_A750	5.3	p.E746_A750		4.4	4.1
MET	CNV	Amplification	4.5*	Amplification		1.8*	1.8*
MYCN	CNV	Amplification	9.5*	Amplification		4.0*	3.6*

*Fold change; SNV, single nucleotide variant; Indel, insertion/deletion; CNV, copy number variant; VAF, variant allele fraction; ng, nanograms.

Table S6. List of genes with variants (frameshift, inframe, missense, stop gain) detected in cfDNA of mCRC patients by TST170.

CRC032	CRC035	CRC062	CRC095	CRC100	CRC106	CRC112	CRC116	CRC131	CRC133
FAM175A	ALK	FAM175A	ALK	FAM175A	ALK	FAM175A	FAM175A	FAM175A	FAM175A
ALK	APC	ALK	APC	ALK	APC	ALK	ALK	ALK	AKT1
APC	ATM	APC	ATM	APC	ATM	APC	APC	APC	ALK
ATM	ATR	ATM	ATR	ATM	ATR	ARID1A	ATM	ATM	APC
ATR	BARD1	ATR	BARD1	ATR	BARD1	ATM	BARD1	ATR	ARID1A
BARD1	BRCA1	BARD1	BRCA1	BARD1	BRCA1	ATR	BCL6	BARD1	ATM
BRCA1	BRCA2	BCL6	BRCA2	BRCA2	BRCA2	BARD1	BRCA1	BRCA1	ATR
BRCA2	BRIP1	BRCA2	BRIP1	BRIP1	BRIP1	BCL6	BRCA2	BRCA2	BAP1
BRIP1	CHEK1	CHEK1	CHEK1	CARD11	CARD11	BRCA1	BRIP1	BRIP1	BARD1
CARD11	CSF1R	CREBBP	EP300	CHEK1	CDKN2A	BRCA2	CHEK1	CHEK1	BRAF
CCNE1	ERBB2	EGFR	ERBB2	EGFR	CHEK1	BRIP1	CREBBP	CTNNB1	BRCA2
CHEK1	ERCC2	EP300	ERBB4	EP300	CSF1R	CCND1	CSF1R	ERBB2	BRIP1
EGFR	EZH2	ERBB2	ERCC2	ERBB2	DNMT3A	CDKN2A	EP300	ERCC2	CDK12
EP300	FANCI	ERCC2	FGF6	FGF23	ERBB2	CHEK1	EZH2	FANCI	CHEK1
ERBB2	FGFR4	FANCL	FGFR4	FGFR2	ERCC2	EP300	FANCI	FGFR4	CREBBP
ERCC2	FLT3	FGF23	FLT1	FGFR4	EZH2	ERBB2	FGF23	GEN1	CSF1R
FANCI	GEN1	FLT3	FLT3	FLT3	FANCI	FANCI	FGFR4	HNF1A	CTNNB1
FGFR4	GNAS	GEN1	GEN1	GEN1	FGF5	FGF6	FLT3	KDR	DDR2
FLT3	HNF1A	HNF1A	IDH1	HNF1A	FGFR4	FGFR3	GEN1	KIT	EP300
GEN1	KRAS	JAK2	KRAS	IDH1	GEN1	FGFR4	HNF1A	KRAS	ERBB2
JAK3	MLH1	JAK3	MLLT3	KMT2A	HNF1A	FLT3	KDR	MLH1	ERBB3
KDR	MSH3	KDR	MSH3	KRAS	KDR	FOXL2	KRAS	MSH3	ERBB4
KRAS	MSH6	KRAS	MUTYH	MLH1	KIT	GEN1	MLLT3	MUTYH	ESR1
MLH1	MYCL1	MLH1	MYC	MSH3	MLLT3	HNF1A	MSH3	MYCL1	FANCI
MSH3	NOTCH3	MSH2	MYCL1	MUTYH	MSH2	KDR	MSH6	NOTCH2	FGF23
MUTYH	PTCH1	MSH3	NBN	MYC	MSH3	KRAS	MUTYH	NOTCH3	FGFR1
MYCL1	PTEN	MUTYH	NOTCH3	MYCL1	MYC	MLH1	MYCL1	PTEN	FGFR2
NBN	RET	MYCL1	PIK3CA	NBN	MYCL1	MLLT3	NBN	RET	FGFR4
NOTCH2	ROS1	NOTCH2	PTCH1	NOTCH1	NRG1	MSH3	NOTCH3	RICTOR	FLT1
NOTCH3	TET2	NOTCH3	PTEN	NOTCH2	PALB2	MYC	NRG1	ROS1	FLT3
NRG1	TP53	PIK3R1	RAD51B	NOTCH3	PDGFRB	MYCL1	PDGFRB	TET2	GEN1
PIK3CD	TSC2	PTCH1	RAD51D	PALB2	PIK3R1	NBN	PIK3CA	TP53	HNF1A
PIK3CG		PTEN	ROS1	PDGFRA	RAD51B	NOTCH3	PTEN		JAK3
PTCH1		RAD51B	TP53	PTCH1	RICTOR	NRG1	RICTOR		KDR
RAD51D		RAD51D	TSC2	PTEN	ROS1	PIK3R1	TET2		KRAS
RB1		RICTOR	XRCC2	RAD51D	SLX4	PTEN	TP53		MDM4
RET		SLX4		RET	SMO	PTPN11			MET
RICTOR		TET2		RICTOR	TP53	RET			MLH1
ROS1		TP53		SLX4	ALK	RICTOR			MRE11A
TET2				SRC	APC	ROS1			MSH3
TP53				TET2	ATM	TET2			MTOR
				TP53	ATR	TP53			MUTYH
					BARD1	TSC1			MYCL1
						TSC2			NBN
									NOTCH1
									NOTCH3
									NRG1
									PALB2
									PIK3CA
									PIK3CG
									PTCH1
									PTEN
									RET
									RICTOR

									ROS1
									SMAD4
									TET2
									TP53
									TSC2

CRC, colorectal cancer patient.

Table S7. List of genes with variants (frameshift, inframe, missense, stop gain) detected in cfDNA of mCRC patients by TST170.

CRC137	CRC142	CRC145	CRC152	CRC154	CRC158	CRC160	CRC164	CRC168
FAM175A	FAM175A	FAM175A	FAM175A	FAM175A	ALK	ALK	FAM175A	FAM175A
ALK	ALK	ALK	ALK	ALK	APC	APC	ALK	AKT2
APC	APC	APC	APC	APC	ARID1A	ATM	APC	ALK
AR	ATM	AR	ARID1A	ATM	ATM	ATR	AR	APC
ATM	ATR	ARID1A	ATM	ATR	ATR	BARD1	ATM	AR
ATR	BARD1	ATM	ATR	BARD1	BARD1	BRCA1	ATR	ATM
BARD1	BCL6	ATR	BARD1	BCL6	BRCA2	BRCA2	BARD1	ATR
BRCA1	BRCA1	BARD1	BRCA1	BRCA1	BRIP1	BRIP1	BRCA2	BARD1
BRCA2	BRCA2	BRCA1	BRCA2	BRCA2	CCNE1	CDKN2A	CHEK1	BRCA1
CCNE1	BRIP1	BRCA2	BRIP1	BRIP1	CD79A	CHEK1	EP300	BRCA2
CHEK1	CHEK1	BRIP1	CHEK1	CARD11	CDK12	EP300	ERBB2	CEBPA
DNMT3A	DNMT3A	CARD11	EP300	CEBPA	CHEK1	ERBB2	ERBB3	CHEK1
ERBB2	ERBB2	CHEK1	ERBB2	CHEK1	CSF1R	FANCI	ERCC2	CSF1R
ERCC2	ERCC2	DDR2	FANCI	CSF1R	DDR2	FGFR4	FANCI	EGFR
FANCI	FANCI	EP300	FGF23	EGFR	DNMT3A	FLT3	FGF23	ERBB2
FGF6	FGF2	ERBB2	FGFR4	ERBB2	EP300	FOXL2	FGFR4	FGF1
FGFR4	FGFR4	FANCI	FLT3	ERBB4	ERBB2	GEN1	FLT3	FGF23
FLT3	FLT3	FGF6	GEN1	ERCC2	ERCC2	HNF1A	GEN1	FGF6
GEN1	GEN1	FGFR4	GNAS	FANCI	EZH2	KDR	GNAS	FGFR4
KMT2A	HNF1A	FLT3	HNF1A	FGF6	FANCI	KRAS	HNF1A	FLT3
MET	JAK3	GEN1	KDR	FGFR4	FGF6	MLLT3	JAK2	GEN1
MLLT3	KDR	KDR	KIT	FLT3	FGFR4	MSH2	KDR	GNAS
MSH3	KIT	MAP2K2	KRAS	GEN1	FLT3	MSH3	KIT	HNF1A
MUTYH	MLH1	MLH1	MLLT3	HNF1A	GEN1	MSH6	KRAS	KDR
MYCL1	MSH3	MLLT3	MSH2	KDR	HNF1A	MUTYH	MLH1	KIT
NRG1	MUTYH	MSH2	MSH3	KIT	IDH1	MYCL1	MLLT3	KRAS
RAD51B	MYCL1	MSH3	MSH6	KMT2A	KRAS	NBN	MSH3	MLLT3
RAD51D	NOTCH3	MUTYH	MYCL1	KRAS	MLH1	NF1	MSH6	MRE11A
RAD54L	NRG1	MYCL1	NBN	MLLT3	MRE11A	NOTCH3	MYC	MSH3
RET	PALB2	NOTCH3	NF1	MRE11A	MSH3	NRG1	MYCL1	MSH6
RICTOR	PIK3CG	PIK3CG	NOTCH1	MSH3	MYCL1	PALB2	NBN	MUTYH
ROS1	PTCH1	PIK3R1	NOTCH2	MSH6	NBN	PDGFRB	NOTCH2	MYCL1
TET2	RAD51B	PTCH1	NOTCH3	MUTYH	NF1	PIK3CA	NOTCH3	NBN
TP53	RAD51D	RAD51B	NRG1	MYCL1	NOTCH3	PIK3R1	NRG1	NOTCH1
XRCC2	RET	RAD51D	PTCH1	NBN	NRG1	PTEN	PTCH1	NOTCH3
	RICTOR	TET2	PTEN	NOTCH3	PDGFRB	RET	PTEN	NRG1
	ROS1	TP53	RAD51D	NRG1	PIK3CA	RICTOR	RET	PIK3CA
	TET2	TSC1	RET	PALB2	PIK3R1	ROS1	RICTOR	PTCH1
	TP53		RICTOR	PIK3CG	PTCH1	TET2	ROS1	PTEN
	TSC1		SMAD4	PTCH1	PTEN	TP53	TET2	RET
	XRCC2		TET2	PTEN	RAD51D	XRCC2	TP53	RICTOR
			TP53	RICTOR	RET		TSC1	ROS1
			VHL	ROS1	RICTOR			SMAD4
				TET2	SLX4			TP53
				TP53	TET2			
				XRCC2	TP53			

CRC, colorectal cancer patient.

Table S8. CNVs detected in cfDNA of patient CRC100 by TST170

Gene	Variant type	Alteration	FC
FGF6	CNV	Amplification	4.59
FGF23	CNV	Amplification	4.93

FC, fold change; CNV, copy number variant.

Table S9. *NRAS* status in cfDNA of mCRC patients analyzed by BEAMIng and TST170.

Sample ID	*NRAS*	
	**BEAMing**	**TST170**
CRC032	WT	WT
CRC035	NA	WT
CRC062	NA	WT
CRC095	WT	WT
CRC100	WT	WT
CRC106	WT	WT
CRC112	NA	WT
CRC116	WT	WT
CRC131	WT	WT
CRC133	WT	WT
CRC137	WT	WT
CRC142	WT	WT
CRC145	WT	WT
CRC152	WT	WT
CRC154	WT	WT
CRC158	WT	WT
CRC160	WT	WT
CRC164	M (VAF: 0.12%)	ND
CRC168	WT	WT

WT, wild type; M, Mutated; VAF, variant allele fraction; NA, not available.

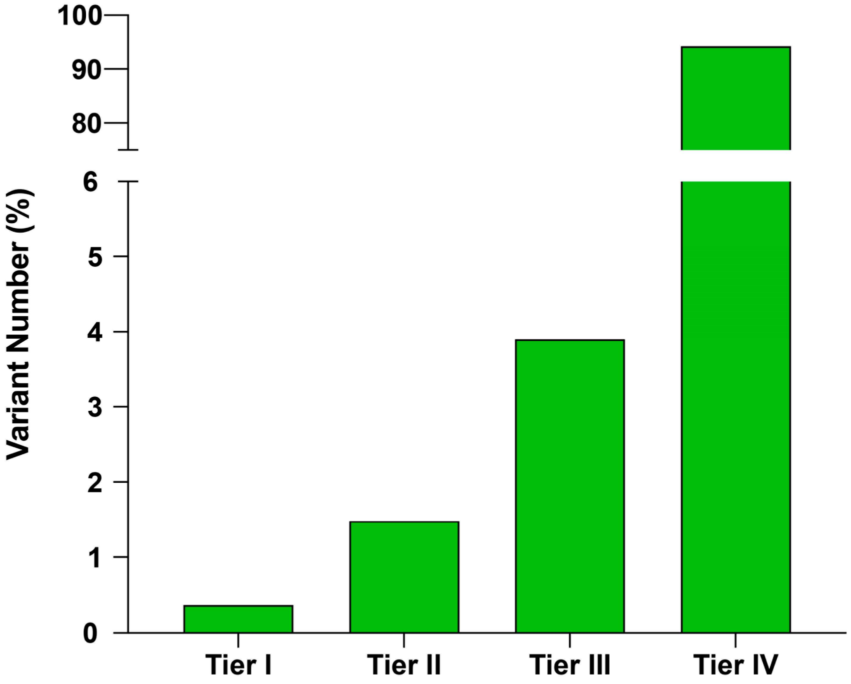

Figure S1. Distribution of variants detected by TST170 in cfDNA of mCRC patients according to their clinical impact. Tier I, variants with strong clinical significance; tier II, variants with potential clinical significance; tier III, variants with unknown clinical significance; and tier IV, benign or likely benign variants.

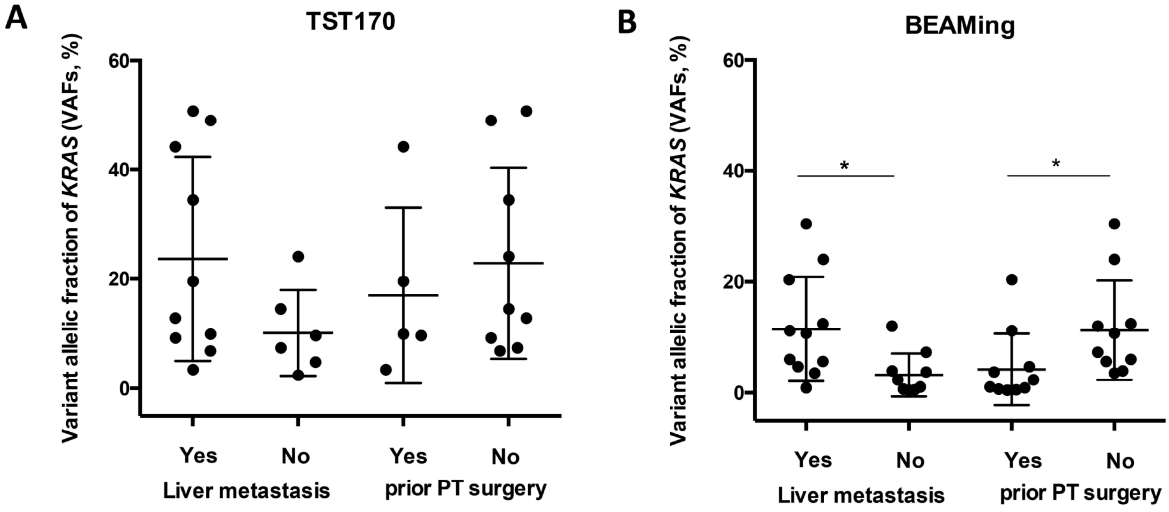

Figure S2. Impact of KRAS VAFs detected by TST170 and BEAMing on patient clinical-pathological characteristics. (A) Levels of KRAS VAFs obtained with TST170 according to metastasis location and previous surgery of primary tumor (PT). (B) KRAS VAFs obtained by BEAMing according to metastasis location and previous surgery for primary tumor (PT). P-values were calculated using Student´s t-test. *$p<0.05$.
